# The role of restraint in fatal excited delirium: a research synthesis and pooled analysis

**DOI:** 10.1007/s12024-020-00291-8

**Published:** 2020-08-22

**Authors:** Ellen M. F. Strömmer, Wendy Leith, Maurice P. Zeegers, Michael D. Freeman

**Affiliations:** CAPHRI School for Public Health and Primary Care, Faculty of Health, Medicine, and Life Sciences, Maastricht University, Maastricht, Netherlands

**Keywords:** Excited delirium, Agitated delirium, Restraint asphyxia, Choke hold, Epidemiology

## Abstract

The purpose of the present study was to perform a comprehensive scientific literature review and pooled data risk factor analysis of excited delirium syndrome (ExDS) and agitated delirium (AgDS). All cases of ExDS or AgDS described individually in the literature published before April 23, 2020 were used to create a database of cases, including demographics, use of force, drug intoxication, mental illness, and survival outcome. Odds ratios were used to quantify the association between death and diagnosis (ExDS vs. AgDS) across the covariates. There were 61 articles describing 168 cases of ExDS or AgDS, of which 104 (62%) were fatal. ExDS was diagnosed in 120 (71%) cases, and AgDS in 48 (29%). Fatalities were more likely to be diagnosed as ExDS (OR: 9.9, *p* < 0.0001). Aggressive restraint (i.e. manhandling, handcuffs, and hobble ties*)* was more common in ExDS (ORs: 4.7, 14, 29.2, respectively, *p* < 0.0001) and fatal cases (ORs: 7.4, 10.7, 50, respectively, p < 0.0001). Sedation was more common in AgDS and survived cases (OR:11, 25, respectively, *p* < 0.0001). The results of the study indicate that a diagnosis of ExDS is far more likely to be associated with both aggressive restraint and death, in comparison with AgDS. There is no evidence to support ExDS as a cause of death in the absence of restraint. These findings are at odds with previously published theories indicating that ExDS-related death is due to an occult pathophysiologic process. When death has occurred in an aggressively restrained individual who fits the profile of either ExDS or AgDS, restraint-related asphyxia must be considered a likely cause of the death.

## Introduction

Excited delirium syndrome (ExDS) is a diagnostic term used to characterize a potentially fatal state of extreme agitation and delirium, often combined with aggressive behavior, tolerance to pain, extreme physical strength and endurance, and hyperthermia [1–4]. The typical presentation of an individual diagnosed with ExDS is a man in his thirties, who 1) died while in a state of agitation and delirium, 2) had a history of drug abuse or mental illness, 3) recently used a stimulant drug such as methamphetamine or cocaine, and 4) was physically restrained at the time of death, most commonly by law enforcement personnel [5]. Because there are no autopsy findings that indicate ExDS, and intoxicant levels in the bodily fluids are typically at a recreational rather than a fatally toxic level, the diagnosis is one of exclusion [6, 7].

The concept of delirium leading to sudden death in an individual who has ingested illicit stimulants was first described in 1985, when the term “Excited Delirium” was used by Wetli and Fishbain to describe six male and one female decedents who exhibited acute agitation, super-human strength, paranoia, mounting fear, hyperthermia, and ultimately cardiorespiratory collapse and death [8]. All of the decedents had non-lethal levels of cocaine in their blood (average 0.6 mg/L), and no anatomic cause of death was found upon autopsy.

Subsequent authors have adopted the term excited delirium and added to or modified the definition and criteria. In 2006 DiMaio and DiMaio coined the phrase “excited delirium syndrome” and described ExDS as an invariably fatal condition that is characterized by the acute onset of delirium with disturbance in consciousness and cognition, combative and violent behavior, physical restraint, and demise due to sudden cardiac death [9]. Some authors have hypothesized that the death is an inexorably fatal process that results from the drugs and agitation, causing a calcium channel blockade in the heart, although no evidence of this phenomenon has been discovered to date [10–12].

When named as a primary cause of death, ExDS is a controversial diagnosis when there is also a history of restraint at the time of death. A major reason for the controversy is the fact that the characteristics that are primarily used to define ExDS (i.e. agitation and delirium) are also highly likely to trigger the use of force and forceful restraint by law enforcement and institutional personnel, and restraint by itself can be associated with an increased risk of death due to positional or compressive asphyxia. Like ExDS, asphyxia is often not associated with any specific pathoanatomical findings at autopsy, and thus the post-mortem examination may provide little guidance in selecting the most likely cause of death. Further complicating the use of ExDS as a cause of death is the fact that the risk factors described in the literature for fatal ExDS completely overlap with the risk factors described for restraint-related asphyxia, including obesity, stimulant drug use, and underlying comorbidities [13–15]. For the preceding reasons, among others, the diagnosis of ExDS in the context of restraint has been posited by some authors as a “cover up” for police excessive use of force, and thus has become a politically charged term [16–18].

Another term that is often used in the literature as a synonym for ExDS is “agitated delirium,” or “agitated delirium syndrome” (AgDS). AgDS is described in the literature using the same terms and risk factors as ExDS, with the exception that the post-mortem/forensic pathology literature almost exclusively uses ExDS (and all cases are fatalities), whereas the clinical medical literature uses both ExDS and AgDS to describe the same pre-mortem patient characteristics, and includes non-lethal as well as lethal cases [19].

Neither ExDS nor AgDS are listed in the International Classification of Diseases (ICD-9, ICD-10), or in the Diagnostic and Statistical Manual of Mental Disorders (DSM-5) [14, 20]. As a result, case descriptions and definitions of ExDS/AgDS are unsystematic, and nearly all of the published research on the topic is limited to retrospective case studies and series [21]. Of the limited studies on groups of individuals diagnosed with ExDS/AgDS, the nature of the study design, small sample sizes, and lack of granularity make it impractical to analyze trends or draw causal conclusions about the diagnoses. A previous systematic review examined definitions, epidemiology, pathophysiology, and management of ExDS, but did not include a statistical analysis of risk factors among the combined cases [21].

There are critical questions surrounding the continued use of the diagnosis of ExDS/AgDS that require further exploration. The first question concerns the potentially confounding effect that restraint has on the risk of death among ExDS/AgDS cases. The second question is more nuanced, but just as critical, which is whether there are characteristics of ExDS that distinguish it from AgDS, aside from the risk of death. If it is found that the only apparent difference between a diagnosis of ExDS versus AgDS is that the victim died *and* was restrained, then the previously advanced theory that a diagnosis of ExDS is confounded by excessive use of force by law enforcement would be supported by the analysis. The finding would also obviate the attempts to explain ExDS deaths via an occult pathophysiologic process. If the evidence indicates that fatal ExDS is what AgDS is called when it results in death during restraint, then the term “ExDS” should be considered an artifact of, rather than an explanation for the death. Investigation of the potential for circular reasoning in how the term ExDS has evolved in the literature and been endowed with a uniquely lethal quality is the primary aim of this paper, as we attempt to evaluate whether there is evidence for ExDS as a unique cause of a death that would have occurred regardless of restraint, or a label used when a restrained and agitated person dies, and which erroneously directs attention away from the role of restraint in explaining the death.

The present analysis is directed at answering these questions. The analysis is thus divided into two steps: the first step is to abstract all individually described case studies of ExDS/AgDS in the scientific literature into a database and analyze the data for associations, and the second step is to collectively describe the grouped data studies in order to evaluate the quality and degree of variability in the literature.

## Methods

A literature search using the National Library of Medicine search engine PubMed, cross-referenced with OVID Medline, resulted in 445 publications that matched the keyword search “excited” or “agitated” and “delirium” as of April 23, 2020, building on the methods used in a previous systematic review of excited delirium [21]. The top 1000 results from the “grey literature” via Google Scholar were cross-referenced, and duplicate studies were consolidated. A total of 1342 studies were reviewed for title and abstract evaluation. Studies were included for a full assessment if they described a case report or case series of ExDS or AgDS, or if the study focused on group characteristics of ExDS/AgDS using aggregated data. Case reports and series describing individual case characteristics were used to create a database of ExDS/AgDS cases, which was then analyzed for trends. Grouped data studies were examined for common characteristics. Although the type of studies that were reviewed did not fit the criteria for a systematic review (as they were case studies and case series), PRISMA guidelines were followed where possible to maximize the quality of the review [22].

**Fig. 1:**
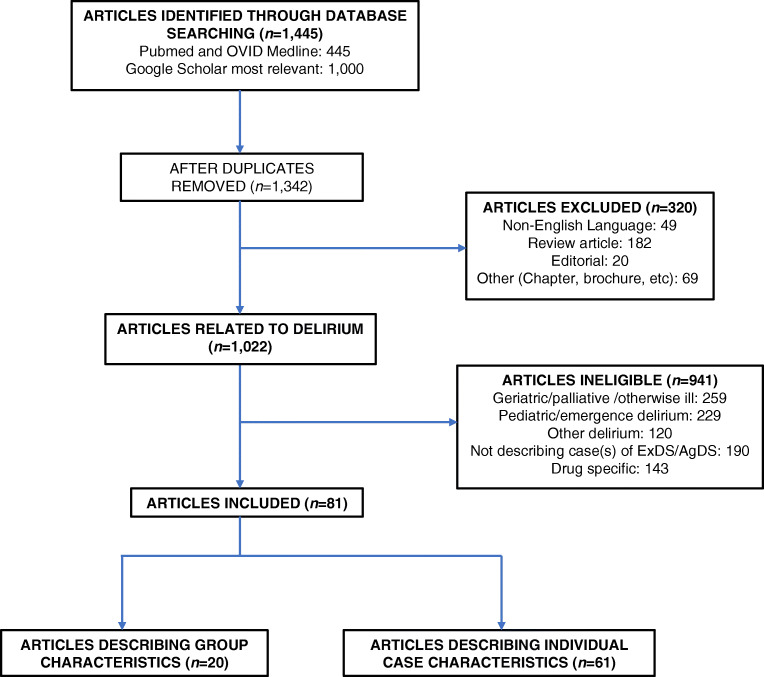
Study selection flowchart

Articles published in a language other than English, review articles, editorials, book chapters, physician brochures, and web pages were excluded from the review. Also excluded were articles related to nonspecific, pediatric, or anesthesia emergence delirium, or that involved geriatric or palliative care, due to the difference in these study populations versus the average population characteristics of ExDS/ AgDS (i.e. previously healthy adults). Studies were also excluded if they described a series or case of agitation or delirium that was deemed neither ExDS nor AgDS, or if the object of the study was reviewing or studying a drug for treatment for undifferentiated delirium (see Fig. 1).

Studies included in the analysis were divided into individual case studies or reports and grouped data studies. Each study and case were reviewed, and available information harvested for the following measures: number of subjects included in the article, diagnosis (ExDS versus AgDS), type of authority (law enforcement, paramedic, etc.), demographics, use of force, drug intoxication, mental health diagnosis, and outcome.

The diagnosis was coded as either excited delirium (ExDS) or agitated delirium (AgDS) based on the terminology used in the article. The type of authority included Police/law enforcement, paramedic, (including health care worker and emergency responder) or other and was based on first arrival to the scene. In instances where police and paramedics were described as arriving at the same time, paramedic was coded because standard emergency response protocol prioritizes paramedic interaction in medical emergencies [23]. Demographics included age, sex, and race. Use of force was coded as none, unknown, manhandle, handcuff, hog/hobble tie, TASER, sedation, and other or unspecified, which included use of pepper spray, 4-point restraints, and blunt objects. Manhandling was defined as use of force or restraint that did not involve handcuffs or other non-manual restraint, and included physical blows to the body, submission or choke holds, tackling the subject to the ground, the use of bodyweight to pin the subject, etc.

Drug intoxication was based on the most commonly described toxicities, and included none, unknown, cocaine, alcohol, marijuana, stimulants (amphetamine, PCP, bath salts, and ecstasy) and other or unspecified. Mental health diagnosis was recorded as none, schizophrenia, bipolar disorder, and other or unspecified. None of the restraint, drug, or mental health variables were mutually exclusive. Outcome was measured as survival or death.

Statistical analyses were performed using SAS software, Version 9.4. An analysis was performed on each variable, for 1) death as an outcome, and 2) the diagnosis (ExDS vs. AgDS). Chi-square was used to evaluate the significance of categorical variables, or Fisher’s exact test for small cell counts, and a two-sample t-test was used for continuous variables, all with a significance of *p* ≤ 0.05.

## Results

Of the 1342 articles screened, 320 were excluded because they were written in a language other than English, or were review articles, editorials, chapters, brochures, or web pages. Of the 1022 remaining articles, 941 were deemed ineligible because they 1) described delirium not diagnosed as ExDS/AgDS or 2) did not describe a case (or group of cases) of ExDS/AgDS. This initial screening process resulted in 81 articles that were included for full review, with 61 papers that described individual cases of ExDS/AgDS and 20 papers that described group characteristics of ExDS/AgDS.

From the 61 articles providing detailed individual information about cases of ExDS or AgDS, 168 individual cases were abstracted and documented. The selected studies and the number of cases contributed by each article are listed in Table 1.

**Table 1:** Articles used to create pooled individual case analysis

Article Author(s)	Year	Number of cases
Aberegg, Erickson, & Cowan [24]	2014	1
Alciati, et al. [25]	1999	3
Atherton, Dye, Robinson, & Beck [26]	2019	2
Benzer, Nejad, & Flood [27]	2013	1
Blaho, et al. [28]	2000	2
Bozeman, Ali, & Winslow [29]	2013	1
Bunai, Azaka, Jiang, & Nagai [30]	2008	1
Burnett, et al. [31]	2012	1
Byard, Cox, & Stockham [32]	2016	1
Byard [5]	2017	1
Corstens [33]	2018	1
Daugherty [34]	2012	2
Debelmas, Benchetrit, Galanaud, & Khonsari [35]	2018	1
Desharnais, et al. [36]	2017	1
Downes, et al. [37]	2015	1
Dyer, Roth, & Hyma [38]	2001	8
Feeney, Vu, & Ani [39]	2010	1
Fishbain & Wetli [40]	1981	1
Ho, et al. [41]	2012	2
Imam, et al. [19]	2013	6
Jovel, Felthous, & Bhattacharyya [42]	2014	1
Kasick, McNight, & Klisovic [43]	2012	2
Kennedy & Savard [44]	2017	1
Kesha, et al. [45]	2013	1
Kiely, Lee, & Marinetti [46]	2009	1
Kodikara, Cunningham, & Pollanen [47]	2012	2
Kowalski, et al. [48]	2017	5
Kristofic, et al. [49]	2016	1
Kunz, Þórðardóttir, & Rúnarsdóttir [50]	2018	1
Labay, et al. [51]	2016	2
Lucena, et al. [52]	2010	3
Lusthof, et al. [53]	2011	1
Maher, Walsh, Burns, & Strote [54]	2014	1
Mash, et al. [55]	2000	8
McDaniel & Miotto [56]	2001	2
Menaker, et al. [57]	2011	1
Miller [58]	1998	1
Mirchandani, Rorke, Sekula-Perlman, & Hood [59]	1994	4
Morrison & Sadler [60]	2001	1
Murphy, Dulaney, Beuhler, & Kacinko [61]	2012	1^†^
Murray, Murphy & Beuhler [62]	2012	1
O’Halloran & Lewman [63]	1993	11
O’Halloran & Frank [64]	2000	20
Park, Korn, & Henderson [65]	2001	2
Penders, Gestring, & Vilensky [66]	2012	3
Pestaner & Southall [67]	2003	2
Plush, et al. [68]	2015	1
Pritchard, DipAnaesth, & Cong [69]	2014	1
Rayamane, et al. [70]	2015	2
Reichmuth, Blanc, & Tagan [71]	2015	1
Ruttenber, McAnally, & Wetli [72]	1999	1
Samuel, Williams, Ferrell [73]	2009	1
Scaggs, Glass, Hutchcraft, & Weir [74]	2016	7
Schiavone, Riezzo, Turillazzi, & Trabace [75]	2016	1
Shields, Rolf, & Hunsaker [76]	2015	1
Śliwicka, Szatner, Borowska-Solonyko [77]	2019	3
Storey [20]	2012	1
Stratton, Rogers, & Green [78]	1995	2
Stratton, Rogers, Brickett, & Gruzinski [14]	2001	18
Sztajnkrycer & Baez [79]	2005	1
Wetli & Fishbain [8]	1985	7
Wiebe, Sigurdson, & Katz [80]	2008	4
**Total individual cases**		168

†The case in this article is described in greater detail in Murray, Murphy & Beuhler (2012), and is therefore omitted from the total count and pooled analysis

The majority of cases (*n* = 161, 95.8%) involved men. Race was missing in most cases (*n* = 95, 56.6%). Law enforcement personnel were the first responders noted on scene in 111 (66.1%) cases. One quarter (*n* = 42, 25.0%) of all events involved a person with at least one known mental illness. The majority of all cases (*n* = 147, 86.3%) had some amount of drug intoxication and some form of use of force (*n* = 138, 81.5%). As a continuous variable, age was not linear, and was categorized into less than or equal to 30 years old, between 30 and 40 years old, and greater than 40 years old for the analysis. Categorization of age was indicated for both the outcome and diagnostic status univariate analyses.

### Analysis of outcome

A univariate analysis of cases by outcome (i.e. fatal or not) was performed on each of the variables. Of the 168 cases, 104 (61.9%) were fatal. Type of authority differed between fatal and survived cases (relative to Law enforcement: paramedic OR: 0.3, *p* < 0.0001; other OR: 0.06, p < 0.0001), as well as diagnosis (ExDS versus AgDS) (OR: 9.9, p < 0.0001). The frequency of drug use was significantly different between the two groups for most of the drug categories: Cocaine, and alcohol were more commonly found in the fatal cases, while marijuana, stimulants, and other drug use were more common in the survived cases. Restraint was documented in 90% of fatal ExDS/AgDS cases, compared with a 68% restraint rate in survived cases. Forceful restraint, such as manhandling, handcuffing, and hog/hobble-tying were all significantly more likely in fatal cases (OR: 7.4, 10.7, 50, respectively, all *p* < 0.0001). See Table 2 for details.

**Table 2:** Characteristics of ExDS and AgDS by death outcome, results from analysis of pooled individual cases

	Total, *n* = 168 (%)	Died, *n* = 104 (%)	Lived, *n* = 64 (%)	Odds Ratio§	*P* value‡
Age					0.009
Less than 30	84 (50.0)	43 (41.4)	41 (64.1)	1 (ref)	
31–40	60 (35.7)	46 (44.2)	14 (21.9)	3.1	
41+	24 (14.3)	15 (14.4)	9 (14.1)	1.6	
**Male**	161 (95.8)	101 (97.1)	60 (93.8)	2.2	0.43
Race					0.71
White	40 (23.8)	22 (21.2)	18 (28.1)	1 (ref)	
Black	23 (13.7)	12 (11.5)	11 (17.2)	1.4	
Other	10 (5.9)	10 (9.6)	0 (0.0)	0.9	
Unknown	95 (56.6)	60 (57.7)	35 (54.7)	>999.9	
Type of authority					**<0.0001**
Police, law enforcement	111 (66.1)	87 (83.7)	24 (37.5)	1 (ref)	
Paramedics	37 (22.0)	7 (6.7)	30 (46.9)	0.3	
Other	20 (11.9)	10 (9.6)	10 (15.6)	0.06	
Diagnosis					
Excited Delirium	120 (71.4)	92 (88.5)	28 (43.8)	9.9	**<0.0001**
Agitated Delirium	48 (28.6)	12 (11.5)	36 (56.3)		
Mental health diagnosis					
None noted	126 (75.0)	81 (77.9)	45 (70.3)	1.5	0.27
Schizophrenia	12 (7.1)	9 (8.7)	3 (4.7)	1.9	0.33
Bipolar disorder	12 (7.1)	9 (8.7)	3 (4.7)	1.9	0.33
Other or Unspecified	22 (13.1)	8 (7.7)	14 (21.9)	0.3	**0.008**
Drug intoxication					
None noted	21 (12.5)	18 (17.3)	3 (4.7)	4.3	**0.02**
Unknown	2 (1.2)	2 (1.9)	0 (0.0)	n/a	0.52
Cocaine	74 (44.1)	55 (52.9)	19 (29.7)	2.7	**0.003**
Alcohol	22 (13.1)	16 (15.4)	6 (9.4)	1.8	0.26
Marijuana	26 (15.5)	5 (4.8)	21 (32.8)	0.1	**<0.0001**
Stimulants^†^	52 (31.0)	28 (26.9)	24 (37.5)	0.6	0.15
Other	42 (25.0)	18 (17.3)	24 (37.5)	0.4	**0.003**
Use of force					
None	3 (1.8)	2 (1.9)	1 (1.6)	1.2	1
Unknown	28 (16.7)	8 (7.7)	20 (31.3)	0.18	**<0.0001**
Manhandle	81 (48.2)	68 (65.4)	13 (20.3)	7.4	**<0.0001**
Handcuff	86 (51.2)	74 (71.2)	12 (18.8)	10.7	**<0.0001**
Hog/Hobble-tie	47 (28.0)	46 (44.23)	1 (1.6)	50	**<0.0001**
Taser	18 (10.7)	14 (13.5)	4 (6.3)	2.3	0.14
Sedation	46 (27.4)	6 (5.8)	40 (62.5)	0.04	**<0.0001**
Other or Unspecified	66 (39.3)	44 (42.3)	22 (34.4)	1.4	0.31

†Stimulants includes amphetamines, PCP, bath salts, and ecstasy

§ Reference categories are indicated by (ref). n/a indicates a zero value and thus OR was incalculable

‡*P* values in bold represent statistical significance at ≤0.05

### Analysis of diagnosis

A univariate analysis of cases by diagnostic status (i.e. ExDS or AgDS) was performed. There were 120 (71.4%) cases of ExDS, compared with 48 (28.6%) cases of AgDS. Outcome (fatal versus non-fatal) was a significant variable in the diagnosis of ExDS, with an OR of 9.9 (*p* < 0.0001). Cases diagnosed as AgDS were more likely to have marijuana and other drug use (*p* = 0.03 and *p* < 0.0001 respectively), while cases diagnosed as ExDS were more likely to involve cocaine, alcohol, or no drug use (*p* = 0.01, 0.03, and 0.04, respectively) The use of manhandling, handcuffs, and hog/hobble tie was more frequent in ExDS cases than in AgDS cases (OR: 4.7, 14, and 29.2 respectively, *p* < 0.0001 for all). Sedation and other or unspecified restraint were more likely to occur in cases diagnosed as AgDS than those diagnosed as ExDS (sedation OR: 0.09, p < 0.0001, other OR: 0.4, *p* = 0.003). See Table 3 for details.

**Table 3:** Characteristics of ExDS cases versus AgDS cases, results from analysis of pooled individual cases

	ExDS *n* = 120 (%)	AgDS *n* = 48 (%)	Odds Ratio§	P value‡
**Outcome death**	92 (76.7)	12 (25.0)	9.9	**<0.0001**
Age				0.32
Less than 30	56 (46.7)	28 (58.3)	1 (ref)	
31–40	47 (39.2)	13 (27.1)	1.8	
41+	17 (14.2)	7 (14.6)	1.2	
**Male**	117 (97.5)	44 (91.7)	3.5	0.09
Race				0.32
White	32 (26.7)	8 (16.7)	1 (ref)	
Black	17 (14.7)	6 (12.5)	0.7	
Other	10 (8.1)	0 (0.0)	0.4	
Unknown	61 (50.8)	34 (70.8)	>999.9	
Type of authority				**<0.0001**
Police, law enforcement	95 (79.2)	16 (33.3)	1 (ref)	
Paramedics	16 (13.3)	21 (43.8)	0.1	
Other	9 (7.5)	11 (22.9)	0.1	
Mental health diagnosis				
None noted	94 (78.3)	32 (66.7)	1.8	0.11
Schizophrenia	10 (8.3)	2 (4.2)	2.1	0.51
Bipolar disorder	9 (7.5)	3 (6.3)	1.2	1
Other or Unspecified	9 (7.5)	13 (27.1)	0.2	**0.0007**
Drug intoxication				
None noted	19 (15.8)	2 (4.2)	4.3	**0.04**
Unknown	2 (1.67)	0 (0.0)	n/a	0.37
Cocaine	60 (50.0)	14 (29.2)	2.4	**0.01**
Alcohol	20 (16.7)	2 (4.2)	4.6	**0.03**
Marijuana	14 (11.7)	12 (25.0)	0.4	**0.03**
Stimulants†	35 (29.2)	17 (35.4)	0.8	0.4
Other	17 (14.2)	25 (52.1)	0.2	**<0.0001**
Use of force				
None	2 (1.7)	1 (2.1)	0.8	1
Unknown	16 (13.3)	12 (25.0)	0.5	0.07
Manhandle	70 (58.3)	11 (22.9)	4.7	**<0.0001**
Handcuff	80 (66.7)	6 (12.5)	14	**<0.0001**
Hog/Hobble-tie	46 (38.3)	1 (2.1)	29.2	**<0.0001**
Taser	16 (13.3)	2 (4.2)	3.5	0.08
Sedation	16 (13.3)	30 (62.5)	0.09	**<0.0001**
Other or Unspecified	39 (32.5)	27 (56.3)	0.4	**0.004**

† Stimulants includes amphetamines, PCP, bath salts, and ecstasy

§ Reference categories are indicated by (ref). n/a indicates a zero value and thus OR was incalculable

‡*P*-values in bold represent statistical significance at ≤0.05

### Analysis of group data studies

The final step of the analysis was to review the grouped data studies, abstract relevant information, and enter it into a spreadsheet. There were 20 grouped data articles, two of which used overlapping data, and so the more comprehensive of the two studies was used for analysis. Of the 19 included articles, 4 papers, totaling 153 cases, used AgDS as a diagnosis, with 5 deaths (3%) and 148 (97%) survivors. The 15 papers that used ExDS as a diagnosis comprised 666 cases and included 529 deaths (79%) and 137 (21%) survivors. One study of in-custody deaths used both diagnoses and reported 19 cases of fatal ExDS and 5 cases of fatal AgDS. One study of 37 subjects used “agitated/excited delirium” as a diagnosis, and was excluded from the comparison between ExDS and AgDS. Only 2 studies used police data to populate their sample, 7 studies used hospital data, and 10 studies used post-mortem sources. The 7 studies that reported low or zero mortality were from hospitals and one from law enforcement. The low or zero mortality studies made up 3 of the 4 AgDS studies. Eight of the 19 articles provided detail about the presence and type of restraint, and the remaining studies either did not mention or did not specify the type of restraint used, so no meaningful conclusions could be drawn regarding the role of restraint in the grouped data studies. See Table 4 for details.

**Table 4:** Data from grouped studies on excited delirium (ExDS) or agitated delirium (AgDS). The author name and year of paper, number of subjects with ExDS or AgDS, data source, and mortality are included

Author	Year	Number of cases		Data source	Type and presence of restraint described	Mortality (%)
		ExDS	AgDS			
Baldwin [1]	2016	73	–	Police data	No	100
Cole [81]	2018	–	49	Inpatient	Yes	0
Ezaki [82]	2016	2	–	Post-mortem	No	100
Grant [83]	2009	21	–	Post-mortem	No	100
Gray [84]	2007	–	31	Emergency dept	No	0
Hall [2, 3]†	2015	86	–	Police data	No	1
Ho [85]	2009	102	–	Post-mortem	Yes	100
Li [86]	2019	31	–	Emergency Dept	No	0
Mash [4]	2009	90	–	Post-mortem	Yes	100
Michaud [87]	2016	35	–	Post-mortem	No	100
Miner [88]	2018	–	68	Emergency dept	No	0
Mo [89]	2020	37		Emergency dept	Yes	0
Pollanen [7]	1998	21	–	Post-mortem	Yes	100
Ross [90]	1998	61	–	Post-mortem	Yes	100
Ruttenber [91]	1997	58	–	Post-mortem	No	100
Southall [15]	2008	19	5	Post-mortem	Yes	100
Strote [92]	2014	43	–	Emergency dept	Yes	100
Strote [93]	2006	3	–	Post-mortem	No	100
Vilke [94]	2019	21	–	Emergency dept	No	0

†The same data used in this 2015 article [2] were included in a 2013 article by the same first author [3]. The later and more comprehensive of the two studies was used for Table 4

## Discussion

The results of the present study indicate that a diagnosis of excited delirium (ExDS) and potentially fatal restraint are inextricably interwoven. Despite the fact that ExDS and AgDS are used interchangeably in the literature and associated with largely identical presentations, [18, 19] the odds of an ExDS diagnosis were nearly 10 times greater than an AgDS diagnosis in the event of a fatality. The most probable mechanism driving the association between ExDS and death is the high frequency of aggressive restraint types observed in the ExDS cases. We found that the most aggressive forms of restraint (i.e. manhandling, handcuffing, and hog/hobble tying) increased the odds of an ExDS diagnosis by between 7 and 29 times, whereas AgDS was 2.5 times more likely to be diagnosed when less aggressive forms of restraint (i.e. pepper spray, 4-point restraint, etc.) were used. Some form of restraint was described in 90% of all deaths, making it the most common factor that is a plausible cause or contributing cause of the death (via asphyxia). Only 2% [*n* = 2] of the fatal cases reported no restraint used, and the remaining 8% [*n* = 8] were unknown or missing. In contrast, 67% [*n* = 43] of survived cases described some form or restraint, 2% [*n* = 1] used no restraint, and 31% [*n* = 20] were unknown or missing, These results provide strong evidence that the more likely it is that a death resulted from restraint, the more likely it is that the death will be attributed to ExDS, which allows for the restraint to be ignored as a cause. Thus, the evidence suggests that ExDS is not a unique cause of death in the absence of restraint, and that the supposition to the contrary is an artifact of circular reasoning and confounding rather than an evidence-based inference. While it is possible that ExDS is a fatal condition in the absence of restraint, there is no observational evidence to support this hypothesis in the biomedical literature at the present time.

**Fig. 2:**
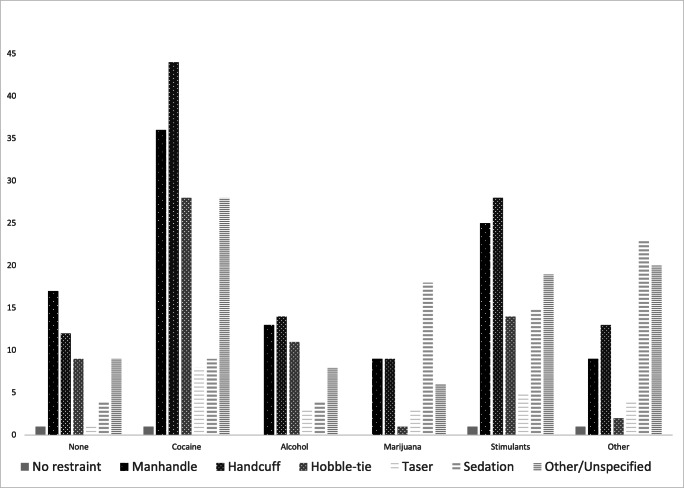
Frequency of restraint type by drug intoxication

An additional interesting result of the analysis of ExDS versus AgDS diagnosis were the findings that “other” drug use, a category that included opiates, mushrooms and LSD, as well as marijuana use, were associated with greater odds of survivability regardless of diagnosis (other/unspecified drug use by 2.5 times, and marijuana by 10 times). Cocaine use, on the other hand, was associated with 2.7 times greater odds of death. A plausible explanation for the protective odds associated with marijuana and opiates etc. is that the effects of these drugs are largely sedating and thus less likely to trigger the use of aggressive restraint methods, whereas the presence of cocaine is more likely to produce combative behavior and thus forceful restraint. This explanation is supported by the associations depicted in Fig. 2, which illustrates the frequency of restraint type stratified by drug type. The chart demonstrates that the most aggressive forms of restraint (i.e. manhandling, handcuffing, and hobble tying) are more commonly associated with the presence of cocaine and other stimulants, and the least aggressive form of restraint (sedation) is most common when marijuana and opiates are present.

An often overlooked fact in the assessment of the cause of death of a forcefully restrained delirious individual with illicit stimulants in their system is that the per-dose risk of death from such drugs is exceedingly low. As an illustration, there were 42 tons of crystal methamphetamine consumed by around 1.2 million people in the US in 2010 [95]. In the same year there were ~ 2700 deaths attributed, at least in part, to methamphetamine exposure, and thus given the typical dose of the drug (10–40 mg), the risk of death from use of the drug is no more than 1 per 353,000 doses [96]. This is not to say that sudden death following recreational illicit stimulant use does not occur sporadically in the absence of other obvious factors, but rather that the presence of the drug should not be preferentially promoted over aggressive restraint as a more probable cause of death in a case of suspected ExDS.

A further piece of evidence supporting the conclusion that fatal ExDS and restraint-related asphyxia are interconnected is the negative association between the date of publication and fatality rate. Before the year 2000, there were 30 cases described in the published literature, 90% of which were fatalities; between the year 2000 and 2020, there have been 138 cases described in the literature, 56% of which were fatal. The explanation for this trend is reasonably attributed to increased awareness of the relationship between aggressive restraint techniques and risk of asphyxia, which lends credence to the supposition that ExDS/AgDS-related death is avoidable in the absence of aggressive restraint. If ExDS-related fatality were purely a pathophysiologic phenomenon distinct from the degree and duration of restraint it would be much less likely that the risk of death would have changed over time for the diagnosis.

Among the reviewed cases the mortality rate associated with a diagnosis of ExDS was 62% for the individual cases and approximately 79% for the aggregate studies. This finding is at odds with what has been previously reported in the literature, most notably by Vilke et al., who stated that ExDS fatality was <10% [18] and by DeBard et al., who stated it was 8.3% [97]. The cited source of the statistic in both papers is a 2007 publication by Barnett, et al. [98]. Problematically, the article, titled “Substance use in a population-based clinic sample of people with first-episode psychosis,” is not about ExDS, nor makes mention of the condition, much less the death rate associated with the diagnosis. Indeed, the paper does not contain any mention of a mortality rate from any cause. The only time the number “8.3” is mentioned in the article is as the standard deviation for the average IQ of study subjects.

In an attempt to demonstrate that restraint is not the most likely cause of death in fatal ExDS, a group of authors published several experimental studies aimed at proving the theory that restraint-related chest compression does not restrict respiration or cardiac output [99–102]. The studies were conducted in controlled environments with healthy volunteer subjects who, in some instances, had up to 210 pounds of weight placed on their back, while they lay prone and hog- or hobble-tied. The authors concluded that there were no clinical (much less potentially fatal) effects from prone restraint regardless of additional weight. They generalized their conclusions to real-world deaths occurring under circumstances of aggressive restraint, casting doubt as to whether any degree of restraint could result in compression-related asphyxia. The authors did not comment on the fact that none of their volunteers were subjected to the typical circumstances in which real-world ExDS deaths occur: likely aggressive restraint applied by multiple law enforcement personnel to a possibly frantic and/or actively resisting individual, who may be intoxicated and already subjected to multiple TASER shocks. Interestingly, the authors responsible for the experimental restraint studies include the same authors who manufactured an artificially low ExDS mortality rate of <10%. The authors fail to note the obvious contradiction in their hypotheses: they claim that ExDS is a condition with a purported fatality rate of <10% due to an unknown inexorable cardiac pathophysiologic event, yet they also claim that addition of chest compression of up to 210 lbs. in such a fragile individual cannot possibly contribute to the risk of death based on an experimental study of chest compression in healthy people.

There are a number of limitations to consider when examining and comparing the ExDS and AgDS literature, and the data that can be abstracted from it. The first is that it is not possible to capture a representative sample of cases of ExDS or AgDS in national hospital databases because neither diagnosis is specified in the ICD-9 or ICD-10, which such databases rely on [103], and the diagnoses are therefore unsearchable in any of the databases. Second, fatal cases are likely overrepresented in the literature because they are more likely to be written about than survived cases. Fatal cases are also more likely to have more detailed information about the circumstances leading to the death because of the nature of death investigation, which typically involves toxicology screens, police reports, and witness accounts. Third, the cases reported in the literature are not a random sample of either AgDS or ExDS, although deaths are likely overrepresented. Fourth, there is often a lack of detail about some of the predictive factors, including which drugs (if any) were used, the quantity and combination of drugs used, what level of tolerance an individual may have had to the involved drugs, and what is considered a drug overdose versus contributory to the death. Fifth, details of restraint are also often missing, including the type, duration, and force of restraint used, where compression may have occurred (i.e. face, neck, chest, appendages), and whether cardiorespiratory collapse occurred during restraint or at a later time.

The variable “manhandling” was used to broadly indicate types of restraint or use of force that may have carried a higher potential for positional asphyxia, and included blows to the body, submission or choke holds, tackling, bodyweight pinning the subject (including from multiple personnel), and other similar actions, which were insufficiently detailed in the majority of the studies for a more granular analysis. It is worthwhile to thus note that not all of the actions included in manhandling for the present analysis carry the same risk of positional asphyxia (i.e. a single blow to the face carries no risk, whereas being pinned to the ground by multiple personnel is relatively high risk). If the cases included in the analysis were more completely described in the literature, such that the low risk manhandling actions could have been excluded from the analysis, the odds of death may have been even greater than the 7.4 times attributed to this category of restraint in the results.

Future study involving a registry in which details of the death are systematically recorded would be ideal. The aforementioned limitations of the literature are bidirectional; while they limit the ability to conclusively state that a majority of ExDS deaths are due to restraint, they also prohibit the inference that the ExDS-related death must be due to anything other than described restraint that directly preceded cardiorespiratory arrest. In other words, there is no reliable evidence to demonstrate that fatal ExDS in the context of restraint is not solely a result of aggressive or forceful restraint in nearly every published case.

In 2009 the American College of Emergency Physicians (ACEP) published a white paper containing the results of an ExDS task force meeting, in which they concluded that the term represents a unique clinical entity, and recommended that the term be adopted for general use in emergency medicine [97]. The white paper authors included the same authors who had published several studies over the preceding decade that purported to demonstrate that asphyxia does not result from virtually any level of restraint-related chest compression up to 210 lbs. [100]. Although the ACEP recommendation was less concerned with fatal ExDS than the management of the agitated and aggressive patient in the emergency department, it lent credibility to the circular reasoning required to believe that fatal ExDS in the context of restraint must involve a hidden lethal mechanism because the death is otherwise unexplainable. The erroneous assumption that restraint-related asphyxia does not have the capacity to alter cardiorespiratory function in an agitated patient such that it can cause death formed part of the rationale behind the promotion, by ACEP, of ExDS as a unique clinical entity that can result in death via an unknown pathophysiologic mechanism unrelated to compressive restraint.

This position is not supported by the literature. At the present time, the data described in the present review are the most comprehensive data available, and the results of this study indicate that ExDS is a diagnostic construct without meaningful clinical or predictive characteristics that distinguish it from AgDS, with the exception of aggressive restraint and associated high risk of death.

A thorough investigation of the circumstances of a death that is suspicious for ExDS/AgDS requires a full autopsy with toxicological analysis, and a thorough review of the circumstances in which the death occurred, including detailed information regarding the type and duration of restraint and force used. Given the fact that there is, at the present time, no apparent literature-based justification for the two separate diagnoses, and the fact that fatal ExDS is, in essence, AgDS with the addition of aggressive and potentially lethal restraint, we suggest that excited delirium be abandoned as a diagnosis in order to avoid systematic error in cause of death determinations. Further, we suggest that agitated delirium replace ExDS in all instances, modified by the degree of restraint used (e.g. AgDS, AgDS + mild restraint, AgDS + aggressive restraint). As an extension of these suggestions, the manner of death for fatal cases of AgDS with a history of restraint would include homicide or undetermined, but not natural or accident. While it is possible that psychosis or drug intoxication alone may have led to an irreversible arrhythmia in the 2 cases of fatal ExDS/AgDS that we reviewed in which no restraint was described (out of 104 deaths), no reliable conclusions can be drawn regarding the cause of these deaths.

## Conclusion

Excited delirium syndrome (ExDS) and agitated delirium syndrome (AgDS) are used interchangeably in the literature, but ExDS is far more likely to be used when the outcome is death and aggressive restraint methods were used. There is no existing evidence that indicates that ExDS is inherently lethal in the absence of aggressive restraint. We recommend that, in the context of medicolegal death investigation, “excited delirium syndrome” be replaced with “agitated delirium syndrome” with a modifier for the presence and degree of restraint used.

## Key points

1.There are no pathoanatomical features that distinguish ExDS from AgDS.2.When a death has occurred and aggressive restraint has been employed, ExDS is diagnosed more frequently than AgDS.3.There is no evidence to support ExDS as a cause of death in the absence of restraint, and only 2% of fatalities did not include a description of some form of restraint.4.In medicolegal death investigation the term ExDS should be abandoned in favor of AgDS, modified by no restraint, mild restraint, or aggressive restraint.
